# An online, peer-mentored food allergy education program improves children’s and parents’ confidence

**DOI:** 10.1186/s13223-023-00800-8

**Published:** 2023-05-29

**Authors:** Ranjit Dhanjal, Kyle Dine, Jennifer Gerdts, Kaitlyn Merrill, Tara Lynn M Frykas, Jennifer LP Protudjer

**Affiliations:** 1 Food Allergy Canada, Toronto, ON Canada; 2 Children’s Hospital Research Institute of Manitoba, Winnipeg, MB Canada; 3 Department of Pediatrics and Child Health, University of Manitoba, Winnipeg, MB Canada; 4 George and Fay Yee Centre for Healthcare Innovation, University of Manitoba, Winnipeg, MB Canada; 5 Department of Foods and Human Nutritional Sciences, Faculty of Agriculture and Food Sciences, University of Manitoba, Winnipeg, MB Canada; 6 Institute of Environmental Medicine, Karolinska Institutet, Stockholm, Sweden

**Keywords:** Mentorship, Education, Food allergy

## Abstract

**Background:**

Children with food allergy, and their families experience substantial burdens because of efforts necessary to minimize the risk of anaphylaxis. To this end, peer-to-peer education is paramount. Food Allergy Canada offers an online, peer-to-peer mentoring program. However, the impact of this program has not previously been formally evaluated.

**Objective:**

To determine if Allergy Pals, an online, peer-to-peer mentoring program, for children aged 7–11 years, increased child and parental food allergy competency, and confidence. Our secondary aim was to qualitatively describe the experiences of the program.

**Methods:**

From May 2020-May 2021, children and their parents were invited to participate in an online, anonymous survey about Allergy Pals, at pre-program, and post-program. Primary outcomes, which were described and compared using chi2 or t-tests, as appropriate for the respective variables, included food allergy competence (epinephrine carriage, signs and symptoms of anaphylaxis) and food allergy confidence (e.g. comfort asking other for food allergy-related support). Secondary outcomes included child and parent perceptions of the program, which were analysed thematically.

**Results:**

Overall, 17 children completed the pre-program, and 11 completed the post-program survey. Corresponding numbers for parents were 25 and 23. Food allergy competence was high pre-program, and remained so post-program. Food allergy confidence improved from pre-program to post-program. E.g. Children tended to feel less left out (5/12, 41.7%; 3/10; 30.0%, respectively), a finding that was reflected also in parents’ scores. Themes identified for child and parent perceptions further supported improved food allergy confidence.

**Conclusion:**

Although food allergy competence was high pre-program, Allergy Pals improved food allergy confidence.

## Introduction

Food allergy is a common childhood condition that affects 7–8% children in industrialized nations [1], including Canada [2]. While promising advancements have been made in therapeutic research, food allergy has no cure and therefore management requires constant vigilance, specifically as it relates to the prevention of anaphylaxis [1]. For these children and their families, this condition is associated with substantial psychosocial [3, 4] and financial burdens [5] resulting from daily management, fear of allergic reactions and projecting future worries. As a result, children with food allergy and their families often have impaired quality-of-life, which appears to increase as affected children age and assume more self-responsibility for their medical condition [6, 7]. Prior to the COVID-19 pandemic, school-aged children with food allergy were often bullied and experienced social exclusion from activities and in settings where food was consumed [1, 8], which decreased during COVID-related school closures [9].

Anaphylaxis risk reduction methods, such as allergen avoidance and food allergy education are key management strategies. To this end, there is a clear need for ongoing education to enhance management and confidence in children, and their families, living with food allergy. Prior to the COVID-19 pandemic, pilot studies provided evidence that food allergy education can improve food allergy knowledge and empathy in those without food allergy, as well as decrease caregiver burden [10–12]. As a result of the COVID-19 pandemic, the rapid shift to virtual learning remains under-evaluated across educational content areas, including food allergy. Despite limited data on the efficiency of virtual education, this format may safely assist food allergic families in managing their child’s condition, and offer them a safe place to express questions and concerns.

Since 2014, the delivery of online educational programs has been a cornerstone at Food Allergy Canada, a not-for-profit national organization that heavily promotes food allergy awareness, education, and advocacy on behalf of Canadians living with food allergy. Allergy Pals, a virtual peer-mentoring program for children ages 7–11 years with food allergies, aims to encourage positive conversations among participants, while providing an opportunity to build relationships and learn from peers’ experiences living with food allergy. The program consists of 8 online sessions, with six children and a peer mentor. To date, however, this program has not been formally evaluated. To this end, we aimed to determine if Allergy Pals increased child and parental food allergy competency, and confidence. Our secondary aim was to qualitatively describe the experiences of the program.

## Methods

### Study population and design

In the present analysis, we restricted the study population to children who had previously taken Food Allergy Canada’s online education programs Allergy Pals (ages 7–11 years) or their parents, and who reported living in Canada.

Between May 2020 and May 2021, participants and their families were invited to participate in an online, anonymous survey about Allergy Pals. Invitations were extended to two groups of participants and parents: pre-program: those who had not yet taken the program, but who were (or whose children were) registered for the following session; and post-program participants: those who had (or whose children had) previously completed Allergy Pals.

***Pre-program*** Three weeks prior to the start of the Allergy Pals session, parents received an introductory email, distributed by Food Allergy Canada, which contained a brief summary of the study and study team. If participants wished to learn more, they clicked on a link to a detailed summary of the study and the study team. If parents agreed to continue to the questionnaire, they clicked on the appropriate link: one for themselves as parents, and one for the child. If participants progressed to the questionnaire, consent was assumed. A total of three emails (the initial contact, plus two reminders, sent with one-week intervals, up to the time the program starts). Once the program started, the link to the survey was no longer valid, and participants were unable to complete the pre-program survey.

***Post-program*** A similar survey was distributed via the same mailing lists one-week post-program. A total of three emails (the initial contact, plus two reminders, were sent with one-week intervals, up to four weeks after the program ended). After four weeks, the link to the survey was no longer valid, and participants were unable to complete the post-program survey. Parents/guardians were not aware that they would be invited to evaluate the program prior to the start of the program or the pre-program.

### Primary outcomes and definitions

**Food allergy competence** included questions on epinephrine carriage to specific events (school, restaurants, sports or clubs, friend’s homes), knowing how to use an EAI (no, yes, not sure), understanding the signs and symptoms of a severe allergic reaction (no, yes, sort of, not sure), and frequency of reading the ingredient list or asking a trusted adult to read the ingredient list (1 = never, 10 = always). If children did not yet consider themselves to be confident readers, they were asked to report how often they asked a trusted adult to read a label on their behalf.

**Food allergy confidence** included questions on comfort reaching out to others (friends, parents, teachers, other) for food allergy related support, with possible closed-ended responses of completely, somewhat, or not at all; feelings of being left out (completely, sometimes, a bit, not at all), friends’ familiarity of using an EAI (no, some do, not sure), comfort asking strangers for help if experiencing an allergic reaction (no, yes, unsure, depends on situation), comfort speaking to others (restaurant staff, teachers, other kids) about food allergy (no, yes, unsure, depends on situation), fear of not being able to effectively deal with an allergic reaction (scale 1–10), and how much food allergy affects interactions with others (scale of 1–10, with 10 indicating the most impact or fear).

### Secondary outcome

Children’s and parents’ reported perceptions upon completion of the program were collected via free text options at the end of the questionnaire.

### Data analysis

***Quantitative data*** were described using n/N, and mean ± standard deviation, and compared using Fisher exact tests (on account of small sample sizes) or t-tests, as appropriate for the respective variables. This analysis was performed using Stata® 17 (College Station, TX). This study was approved by the University of Manitoba Health Research Ethics Board (HS23323 (H2019-415). We hypothesized that both food allergy competence and confidence would change from pre-program to post-program, with p ≤ 0.05 defined as being statistically significant.

***Qualitative data*** were analysed via thematic analysis, an approach that permits description of responses, including what was reported, and by whom, and with what effect [13]. Frequency of the most common words have been reported with verbatim quotations [corrected for spelling, generic name substituted for product name] to support themes.

## Results

### Demographics

In total, 17 children completed the pre-program, and 11 completed the post-program survey (Table 1). Corresponding numbers for parents were 25 and 23. Amongst children, gender representation was comparable for both data collection points, whereas parents of boys predominated for parent-reported data. The majority of participants lived in Ontario, the most populous province in Canada. Across groups, allergies to peanut and tree nut predominated, and the majority of children reported that they recognized that they had food allergy between ages 3–5 years.

**Table 1:** Characteristics of Children and Parents

	Children				Parents			
	Pre-program (N = 17)		Post-program (N = 11)		Pre-program (N = 25)		Post-program (N = 23)	
	n	%	N	%	n	%	n	%
Boys	9	56.3	5	50.0	15	60.0	16	69.6
Ontario residence	9	52.9	8	80.0	12	48.0	15	65.2
Food allergies*								
Milk	1	5.9	1	9.1	2	8.0	3	13.0
Egg	0	0.0	2	18.2	2	8.0	2	8.7
Peanut	14	82.4	10	90.9	19	76.0	19	82.6
Tree nut	13	76.5	9	81.8	20	80.0	19	82.6
Fish	3	17.6	0	0.0	2	8.0	2	8.7
Crustaceans	4	23.5	4	36.4	8	32.0	5	21.7
Soy	2	11.8	1	9.1	1	4.0	2	8.7
Wheat and Triticale	0	0.0	1	9.1	0	0.0	1	4.3
Sesame	5	29.4	4	36.4	6	24.0	7	30.4
Mustard	0	0.0	0	0.0	0	0.0	0	0.0
Other†	5	29.4	1	9.1	5	20.0	4	17.4
Age at Diagnosis‡								
0–2 years	6	35.3	3	27.3	21	84.0	0	0.0
3–6 years	10	58.8	8	72.7	4	16.0	3	13.0
7–11 years	1	5.9	0	0.0	0	0.0	0	0.0

*Not mutually exclusive

†Includes buckwheat, oats, barley, mango, fruit, vegetables sunflower seeds, pumpkin seeds, hemp seeds

‡For participants, this refers to age at which they recognized that they had food allergy

### Food allergy competence

Amongst children and parents, EAI carriage and knowledge of use, and anaphylaxis recognition was high pre-program and post-program (Table 2). Of note, fewer children reported EAI carriage to sports or clubs after taking Allergy Pals, albeit, at p = 0.056, a difference that did not quite reach statistical significance. Both children and parents reported that children regularly read ingredient lists or asked a trusted adult to do so (e.g. children: pre-program 8.5 ± 2.6/10; post-program 8.7 ± 1.6/10). Importantly, a higher proportion of both children and parents reported recognizing the signs and symptom of anaphylaxis post-program vs. pre-program (children: 100.0% vs. 68.8%, p = 0.06; parents: 95.7% vs. 56.0%, p < 0.01).

**Table 2:** Pre- and post-program food allergy competence, of children and parents

	Children							Parents						
	Pre-program			Post-program			Pre-program				Post-program			
	n	N	%	n	N	%	n		N	%	n	N	%	
**Always carry EAI**														
School	15	16	93.8	10	11	90.1	25		25	100.0	23	23	100.0	
Restaurants	16	16	100.0	9	11	81.8	21		25	84.0	23	23	100.0	
Sports or clubs	16	16	100.0	8	11	72.7	22		25	88.0	23	23	100.0	
Friend’s home	15	16	93.8	8	11	72.7	24		25	96.0	23	23	100.0	
**Know how to use EAI**	13	16	81.3	8	11	72.7	22		25	88.0	21	23	91.3	
**Signs and symptoms of anaphylaxis**	11	16	68.8	11	11	100.0	14		25	56.0	22*	23	95.7	
**Frequency of reading ingredient list or asking trusted adult to read ingredient list†**														
	15	8.5 ± 2.6		11	8.7 ± 1.6		24		7.7 ± 2.6		23	8.3 ± 2.2		

* Significant improvement (p < 0.01, compared to corresponding pre-program competence)

†Scale from 1 to 10, where 1 = never, and 10 = always

### Food allergy confidence

Compared to pre-program, fewer children reported being “somewhat comfortable” (70.6% vs. 27.3%, p = 0.05) reaching out to friends for food allergy-related support, whereas more children reported being “completely comfortable” (Table 3). Most children were “somewhat comfortable” reaching out to parents pre-program, which persisted to post-program (15/6, 93.7% vs. 9/10, 90.0%, respectively). Parent scores were on a scale of 1–10, and did not differ significantly between pre-program and post-program (all p > 0.14). Compared to pre-program, parents reported that their children felt less completely left out, albeit which did not quite reach statistical significance (3.9 ± 2.8 vs. 5.4 ± 2.7, respectively; p < 0.07).

**Table 3:** Pre- and post-program food allergy confidence, of children and parents

	Children						Parents											
	Pre-program			Post-program			Pre-program						Post-program					
	n	N	%	n	N	%	n		N		%		n		N		%	
**Comfort level reaching out to others for food allergy-related support**																		
Friends							25		7.3 ± 2.7				22		7.0 ± 2.5			
Completely comfortable	1	17	5.9	4	11	36.4	-		-				-		-			
Somewhat comfortable	12	17	70.6	3*	11	27.3	-		-				-		-			
Parents							25		8.8 ± 1.9				21		8.3 ± 2.1			
Completely comfortable	1	16	6.3	1	10	10.0	-		-				-		-			
Somewhat comfortable	15	16	93.7	9	10	90.0	-		-				-		-			
Teachers							25		7.4 ± 2.7				21		8.4 ± 1.6			
Completely comfortable	13	16	81.3	6	11	54.6	-		-				-		-			
Somewhat comfortable	3	16	18.7	5	11	45.4	-		-				-		-			
Others							20		6.8 ± 3.0				22		7.0 ± 2.5			
Completely comfortable	5	12	41.7	2	11	18.2	-		-				-		-			
Somewhat comfortable	5	12	41.7	7	11	63.6	-		-				-		-			
Completely feel left out**, ^**†**^	5	12	41.7	3	10	30.0	25		3.9 ± 2.8				22		5.4 ± 2.7			
**Some friends know how to use EAI**	13	16	81.3	8	11	72.7	13		24		54.2		4		23		17.4	
**Comfort levels…**																		
Asking strangers if experiencing reaction	6	15	40.0	8	11	72.7	0	24		0.0		16*		23		69.6		
Speaking to:																		
Restaurants																		
Completely comfortable	5	15	33.3	1	11	9.1	0	24		0.0		7*		23		30.4		
Somewhat comfortable	10	15	66.7	10	11	90.9	9	24		37.5		12		23		52.2		
Teachers																		
Completely comfortable	4	15	26.7	0	11	0.0	4	24		16.7		8		23		34.8		
Somewhat comfortable	10	15	66.7	11	11	100.0	5	24		20.8		14*		23		60.9		
Other Kids																		
Completely comfortable	5	15	33.3	1	11	9.1	3	24		12.5		9*		23		39.1		
Somewhat comfortable	9	15	60.0	8	11	72.7	10	24		41.7		12		23		52.2		
**Fear of not being able to deal effectively with an allergic reaction†§**																		
	15	6.87 ± 2.38		11*	7.8 ± 3.2		24	8.1 ± 1.8										
**How much food allergy affects interactions with others§**																		
	15	6.87 ± 2.39		11	3.2 ± 2.0		24	5.9 ± 2.8				22		6.1 ± 2.3				

* Significant improvement (p ≤ 0.05, compared to corresponding pre-program competence)

†Scale from 1 to 10, where 1 = never, and 10 = always

‡Not queried of parents post-program

§Scale from 1 to 10, where 1 = always, and 10 = not at all.

**Children’s feelings of being left out: preprogram n corresponds to feeling left out at least sometimes; post-program n corresponds to those feeling left out in similar ways as pre-program

Compared to pre-program, children reported that food allergy had less effect on their interactions with others, post-program (6.87 ± 2.38 vs. 3.2 ± 2.0, p = 0.004). Whereas parents did not report any statistically significantly differences in these interactions, compared to pre-program, at post-program, parents noted that their children would be significantly more comfortable asking a stranger for help if the child was experiencing an allergic reaction (p < 0.001), that more children were completely comfortable speaking to restaurants (p = 0.004), somewhat comfortable speaking to teachers (p = 0.006) and completely comfortable speaking to their friends (p < 0.05) about food allergy.

### Qualitative findings

Two distinct themes were identified from children’s open-text descriptions of the program, whereas three themes were identified from parents’ responses (Table 4). The theme with the highest frequency of common words was identified for the parent theme, “My child doesn’t feel so alone.” This theme, which reflects that children gained knowledge that they were not alone in their food allergy experience, was captured in the parent quote:

Connecting her with other youth and people with food allergies so she doesn’t feel so alone in her food allergies and also being inspired by some of the things that others with food allergies have done to bring about awareness around food allergies.

**Table 4:** Qualitative content analysis of children’s and parents’ experiences post-program

Theme	Meaning	Supporting Quotations	Frequency of common words
**Children**			
**Knowledge gives me more freedom**	With greater knowledge, children feel more confident managing their food allergy to gain new experiences.	*“What to do in a restaurant because I’ve never been to one”* *“[L]earning how to view my food allergy as something positive”*	Auto-injector (n = 4)
**I made friends who understand my allergy**	Being involved in the program gave children an opportunity to connect with others	*“[M]eeting friends with allergies and hearing their stories”* *“Liked speaking to others with allergies.”*	Speaking, hearing (n = 2) Meeting (n = 1)
**Parents**			
**By connecting with others, my child is more empowered to deal with their food allergy**	Parents perceived that participation in the program increased their children’s food allergy competence and confidence	**Competence** ***“*** *Become more aware of her anaphylactic symptoms and importance of [EAI]”* **Confidence** *“To not be shy to speak up and ask questions”* *“confident communication skills and empathizing with others”*	Aware (n = 3) Speak up, communicate, talk (n = 5)
**Tolerance for risk is not one size fits all**	Parents appreciated that their children internalized that each family has a difference in risk tolerance	*“That each family has a different threshold for what is acceptable and what is not acceptable as it relates to food allergies - i.e. dining out in our family vs dining out in someone else’s family might be at completely different comfort levels”*	Threshold (n = 1)
**My child doesn’t feel so alone**	Children learned that they had a support network of peers from across the country who shared similar food allergy-related lived experiences	*“he has a support network who really “understands” how he feels and what he goes through every day”* *“My child learned that there is support for him from non-family members, and that other children share his experiences with allergy.”* *“Connecting her with other youth and people with food allergies so she doesn’t feel so alone in her food allergies and also being inspired by some of the things that others with food allergies have done to bring about awareness around food allergies.”* *“That he’s not alone - there are other people across the country that share his allergy concerns/experiences and that he can communicate with.”*	Not alone (n = 10) Connect (n = 4) Support (n = 3) Kids, friends, children (n = 8)

Abbreviation: *EAI* epinephrine autoinjector

## Discussion

In this evaluation of food allergy competence and confidence prior to, and following completion of an eight-week online, peer-mentorship program designed for children ages 7–11 years living with food allergy, we identified that food allergy competence was high at the start of the study, and thus changed little over the course of the program. In contrast, both children and their parents reported that food allergy confidence improved quantitatively and qualitatively.

Food allergy competence remained stable, both quantitatively and qualitatively, from pre-program to four weeks’ post-program, even though Allergy Pals did include content on food allergy education. Yet, this null finding must be considered in the context of the study population. To participate in Allergy Pals, children needed to be ages 7–11 years. All children had lived with food allergy for several years, including one-third (35.3%) who had been diagnosed as infants. As such, both children and their parents likely began Allergy Pals with high food allergy competence. This was reflected in both pre-program reports of EAI carriage, knowledge how to use an EAI, and to reading of ingredient labels both at home and outside the home. More broadly, this may be reflected by a positive relationship between health literacy and self-reported involvement in patient organisations [14]. Indeed, as most people generally eat, at minimum, daily, the ability to effectively manage food allergy is a core competence to prevent a potentially fatal allergic reaction. In this light, we posit that food allergy competence must receive focus immediately following diagnosis. At the same time, non-statistically significant decreases in EAI carriage post-program provide evidence that food allergy competence must receive sustained focus. In contrast, confidence may require time to develop.

Competence and confidence remain distinct constructs. While literature from other diseases supports that low competence may be associated with poorer mental health outcomes and health behaviours, much of this research, to date, has focused on conditions that present in later life [15, 16] or include patients across diverse age groups [17]. There is a paucity of literature on food allergy competence in young children and their parents. However, based on the literature from other medical conditions [15–17], we believe that increased competence may contribute to increased confidence in the management of food allergy, with some caveats.

Food allergy is a rapidly evolving field, with substantial changes to practice reported in the past decade, of which the most notable are arguably the revised guidelines regarding early introduction [18] and an increased demand for oral immunotherapy [19]. In this light, it is essential that families managing food allergy have access to current, evidence-based guidance for food allergy management, so they are able to maintain their existing competence. Juxtaposed against the rapid evolution of food allergy is the use of social media for food allergy information [20], misinformation [21], and inappropriate) portrayal of food allergy [22], all of which have the potential to negatively impact food allergy confidence.

In our study, an online, anonymous food allergy education program moderated by peers, not healthcare professionals, yielded substantial and some significant improvements in food allergy confidence. Elsewhere, multidisciplinary healthcare professionals teams have been described as requires supports to improve mothers’ food allergy confidence [23]. Yet, owing to a paucity of food allergy health professionals across Canada [24], peer mentorship may play an important role in improving confidence while minimizing any additional burden to food allergy health professionals. Encouragingly, an American mentorship program for parents whose children age < 5 years had been diagnosed in the past year yielded similarly favorable results, including improved food allergy confidence [25]. Likewise, a Spanish parent mentorship program, co-developed by healthcare professionals, researchers and patient partners highlighted the benefits of sharing experiences when navigating a food allergy diagnosis [26].

Food allergy management is a shared responsibility. Thus, the need for children to develop confidence to self-advocate and recognise their responsibility is critical. The children in our study were ages 7–11 years. This may be an appropriate age for children to being working with peer mentors, as it is the age at which children with food allergy recognise that their condition is associated with poorer mental health outcomes, particularly within the domains of food allergy anxiety and emotional impact [27].

We acknowledge the limitations of our study, most notable of which was the small sample size. However, this may be in part, reflective of the small group sizes for Allergy Pals. As this was an anonymous survey distributed to the parents/guardians of all participants in the Allergy Pals program, we have no possible way of linking the pre- and post-program surveys.

Notable strengths of the study include the mixed methods design. Collectively, the quantitative and qualitative findings provide a more complete understanding of the benefit of the program [28]. Moreover, we collected data from both children in the program and from their parents, via independent online surveys.

In conclusion, while competency in food allergy management is a necessary foundation, quality of life for children with food allergy is realized by being able to confidently advocate for their needs. The Allergy Pals program reinforced for participants that they are not alone and others in the same situation also need to advocate. At the end of an online, peer-mentored food allergy education program, participating children reported greater food allergy confidence, which was echoed through parent reports. This confidence was attributed to greater feelings of empowerment, self-advocacy and community, and reduced feelings of isolation.

## Data Availability

Written requests for anonymous data will be considered by the authors.

## Abbreviations

EAI: Epinephrine autoinjector
OIT: Oral immunotherapy
PAL: Precautionary allergy labels

## References

[1] Sicherer SH, Sampson HA (2018). Food allergy: a review and update on epidemiology, pathogenesis, diagnosis, prevention, and management. J Allergy Clin Immunol.

[2] Clarke AE, Elliott SJ, St Pierre Y, Soller L, La Vieille S, Ben-Shoshan M (2020). Temporal trends in prevalence of food allergy in Canada. J Allergy Clin Immuonol Pract.

[3] Golding MA, Gunnarsson NV, Middelveld R, Ahlstedt S, Protudjer JLP (2021). A scoping review of the caregiver burden of pediatirc food allergy. Ann Allergy Asthma Immunol.

[4] Golding MA, Batac ALR, Gunnarsson NV, Ahlstedt S, Middelveld R, Protudjer JLP (2022). The burden of food allergy on children and teens: a systematic review. Pediatr Allergy Immunol.

[5] Fong AT, Ahlstedt S, Golding MA, Protudjer JLP (2022). The economic burden of food allergy: what we know and what we need to learn. Curr Treat Options Allergy.

[6] Protudjer JLP, Jansson SA, Middelveld RJM, Östblom E, Dahlén S-E, Heibert Arnlind M et al. Impaired health-related quality of life in adolescents with allergy to staple foods. Clin Trans Allergy. 2016;6(37). 10.1186/s13601-016-0128-5 PMC5045620 27733903

[7] Thörnqvist V, Middelveld R, Wai HM, Ballardini N, Nilsson E, Strömquist J et al. Health-related quality of life worsens by school age amongst children with food allergy. Clin Trans Allergy. 2019. 10.1186/s13601-019-0244-0 PMC6366088 30774928

[8] Fong AT, Katelaris CH, Wainstein BK (2018). Bullying in australian children and adolescents with food allergies. Pediatr Allergy Immunol.

[9] Merrill KA, Abrams EM, Simons E, Protudjer JLP (2020). Social well-being amongst children with vs. without food allergy before and during COVID-19. Ann Allergy Asthma Immunol.

[10] Gillespie C, Ross N, Cisneros NF, Becker AB (2014). Development and piloting of a food allergy education program for parents of young children. J Allerg Clin Immunol.

[11] Polloni L, Baldi I, Lazzarotto F, Bonaguro R, Toniolo A, Gregori D (2020). Multidisciplinary education improves school personnel’s self-efficacy in managing food allergy and anaphylaxis. Pediatr Allergy Immunol.

[12] Yamamoto-Hanada K, Honda T, Kurihara J, Ishitsuka K, Futamura M, Ohya Y (2016). Food allergy education program at an elementary school: a pilot study. Ann Allergy Asthma Immunol.

[13] Bloor M, Wood F (2006). Keywords in qualitative methods: a vocabulary of research concepts.

[14] Brabers AEM, Rademakers JJDJM, Groenewegen PP, van Dijk L, de Jong JD (2017). What role does health literacy play in patients’ invovlement in medical decision making?. PLoS ONE.

[15] Bachman JM, Goggins K, Nwosu S, Schildcrout JS, Kripalani S, Wallston KA. Perceived health competence predicts health behavior and health-related quality of life in patients with cardiovascular disease. Patient Educ Counsel. 2016;99(12). 10.1016/j.pec.2016.07.020 PMC5525151 27450479

[16] Xie A, Du J, Liu Y, Li Z. Perceived health competence and health education experience preduct health promotion behaviors among rurla older adults: a cross-sectional study. BMC Public Health. 2022;22(16479). 10.1186/s12889-022-14080-1 PMC9442915 36064340

[17] Leslie CE, Schofield K, Vannatta K, Jackson JL. Perceived health competence predicts anxiety and depressive symptoms after a three-year follow-up among adolescents and adults with congential health disease. Eur J Cardiovasc Nurs. 2019;19(4) 10.1177/1474515119885858 PMC13034467 31722548

[18] Abrams EM, Hilderand K, Blair B, Chan ES (2019). Timing of introduction of allergenic solids for infants at high risk. Paediatr Child Health.

[19] Perrett KP, Sindher SB, Bégin P, Shanks J, Elizur A (2022). Advances, practical implementation, and unmet needs regarding oral immunotherapy for food allergy. J Allergy Clin Immunol Pract.

[20] Alvarez-Perea A, Cabrera-Freitag P, Fuentes-Aparicio V, Infante S, Zapatero L, Zubeldia JM (2018). Social media as a tool for the management of food allergy in children. J Invest Allergol Clin Immunol.

[21] Reddy K, Kearns M, Alvarez-ARango S, Carrillo-Martin I, Cuervo-Pardo N (2018). YouTube and food allergy: an appraisal of the educational quality of information. Pediatr Allergy Immunol.

[22] Abo MM, Slater MD, Jain P (2017). Using health conditions for laughs and health policy support: the case of food alleries. Health Commun.

[23] Aika S, Ito M, Yamamoto Y (2017). Food allergy response capabilities of mothers and related factors. Nurs Health Sci.

[24] Canadian Medical Association. Clinical Immunology / Allergy Profile. (last updated. December 2019). Available at https://www.cma.ca/sites/default/files/2019-01/immunology-allergy-e.pdf on 20221115.

[25] Ramos A, Cooke F, Miller E, Herbert L (2021). The Food Allergy parent mentoring program: a pilot intervention. J Pediatr Psychol.

[26] Ruiz-Baqués A, Contreras-Porta J, Marques-Mejias M, Cárdemas Rebollo JM, Capel Torres F, Ariño Pla MN (2018). Evaluation of an online educational program for parents and caregivres of children with food allergies. J Invest Allergol Clin Immunol.

[27] Thörnqvist V, Middelveld R, Wai HM, Ballardini N, Nilsson E, Strömquist J, Ahlstedt S, Nilsson LJ, Protudjer JLP. Health-related quality of life worses by school age amongst children with food allergy. Clin Trans Alergy. 2019;9(10). 10.1186/s13601-019-0244-0 PMC6366088 30774928

[28] Creswell JW, Plano-Clark VL (2007). Designing and conducting mixed methods research.

